# Online Monitoring of Electrochemical Carbon Corrosion in Alkaline Electrolytes by Differential Electrochemical Mass Spectrometry

**DOI:** 10.1002/anie.201909475

**Published:** 2019-12-04

**Authors:** Sandra Möller, Stefan Barwe, Justus Masa, Daniela Wintrich, Sabine Seisel, Helmut Baltruschat, Wolfgang Schuhmann

**Affiliations:** ^1^ Analytical Chemistry—Center for Electrochemical Sciences—CES Faculty of Chemistry and Biochemistry Ruhr University Bochum Universitätsstrasse 150 44780 Bochum Germany; ^2^ Institute for Physical and Theoretical Chemistry University of Bonn Römerstrasse 164 53117 Bonn Germany

**Keywords:** carbon corrosion, cell design, DEMS, differential electrochemical mass spectrometry, electrocatalysis

## Abstract

Carbon corrosion at high anodic potentials is a major source of instability, especially in acidic electrolytes and impairs the long‐term functionality of electrodes. In‐depth investigation of carbon corrosion in alkaline environment by means of differential electrochemical mass spectrometry (DEMS) is prevented by the conversion of CO_2_ into CO_3_^2−^. We report the adaptation of a DEMS system for online CO_2_ detection as the product of carbon corrosion in alkaline electrolytes. A new cell design allows for in situ acidification of the electrolyte to release initially dissolved CO_3_^2−^ as CO_2_ in front of the DEMS membrane and its subsequent detection by mass spectrometry. DEMS studies of a carbon‐supported nickel boride (Ni_*x*_B/C) catalyst and Vulcan XC 72 at high anodic potentials suggest protection of carbon in the presence of highly active oxygen evolution electrocatalysts. Most importantly, carbon corrosion is decreased in alkaline solution.

## Graphical abstract

**Caught in the act**: Direct detection of CO_2_ as a marker for carbon oxidation in alkaline electrolytes is now possible using a novel DEMS cell (DEMS=differential electrochemical mass spectrometry) and a specially designed experimental procedure. A high oxygen evolution reaction (OER) catalyst loading on carbon suppresses carbon corrosion in alkaline media.

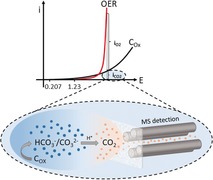

Carbon materials in their various allotropic forms, as bulk materials (e.g. graphite and glassy carbon), powders (e.g. carbon nanotubes and graphene), carbon fibers, carbon foils and pastes, among others, are extensively employed in electrochemical technologies. In electrocatalysis, carbon is used as support for dispersion of precious‐metal catalyst nanoparticles to enhance their utilization, and as a conductive matrix to boost charge transfer of inherently low‐conductivity catalysts.^1^ Recent developments of so‐called heteroatom‐doped carbon catalysts or catalyst supports, for example, nitrogen‐, boron‐, or phosphorus‐doped carbon, has revealed interesting new applications of such carbon‐based materials as noble‐metal‐free catalysts for the oxygen reduction reaction (ORR),^2^ the oxygen evolution reaction (OER),^3^ and the CO_2_ reduction reaction (CO_2_RR),^4^ to name but a few. A core concern of using glassy carbon electrodes^5^ and carbon as an electrode material, catalyst, or catalyst support in electrochemical systems in general relates to its susceptibility to corrode under oxidizing conditions^6^, ^7^ through dissolution, gasification, or exfoliation under formation of corrosion products that affect the carbon properties. In the past three decades, studies on carbon corrosion predominantly focused on acidic electrolytes,^8^, ^9^, ^10^ mainly because of the broad research interest in proton exchange membrane fuel cells (PEMFCs) and electrolyzers. Carbon corrosion was intensively studied using various analytical techniques, including Raman spectroscopy,^11^ FT‐IR spectroscopy,^12^ X‐ray diffractometry,^11^ X‐ray photoelectron spectroscopy,^13^ and identical location transmission electron microscopy.^14^ Carbon becomes thermodynamically unstable at potentials higher than its equilibrium potential of 0.207 V versus reversible hydrogen electrode (RHE).^15^ The consequences of carbon corrosion typically include a decrease of the electrochemically active surface area (ECSA) as well as the conductivity. Electrochemical oxidation of carbon leads to the formation of both soluble and insoluble organic and inorganic products in the electrolyte. Typical products of carbon electrooxidation include CO and CO_2_,^6^, ^16^, ^17^ [Eqs. (1), (2)).

(1) $$C{}_{\left(s\right)}+H{}_{2}O\to \mathrm{CO}+2\;H{}^{+}+2\;e{}^{-}\;\left(E{}^{0}=0.518\;V{}_{\mathrm{SHE}}\right)$$

(2) $$C{}_{\left(s\right)}+2\;H{}_{2}O\to \mathrm{CO}{}_{2}+4\;H{}^{+}+4\;e{}^{-}\;\left(E{}^{0}=0.207\;V{}_{\mathrm{SHE}}\right)$$

CO formation is thermodynamically hindered due to its high standard potential, while CO oxidation to CO_2_ is favored [Eq. (3)] with a standard potential of *E* ^0^=−0.103 V_SHE_.

(3) $$\mathrm{CO}{}_{\left(g\right)}+H{}_{2}O\to \mathrm{CO}{}_{2}+2\;H{}^{+}+2\;e{}^{-}\;\left(E{}^{0}=-0.103\;V{}_{\mathrm{SHE}}\right)$$

In contrast to soluble inorganic and insoluble organic products of carbon oxidation, such as graphite oxides and surface oxygen functional groups (C=O, C−O−C and O−C=O),^18^ soluble organic products, such as mellitic and humic acids, are formed at very low concentrations and therefore considered insignificant.^19^ Studies show that carbon materials with a high degree of graphitization, such as carbon nanotubes and graphene, exhibit comparatively superior corrosion resistance as compared to amorphous carbon.^9^, ^10^, ^20^ Suppressing carbon corrosion in electrochemical applications is therefore of crucial importance. Carbon oxidation, as well as the underlying corrosion mechanisms has been widely investigated in acidic electrolytes.^10^, ^17^, ^21^ In contrast, studies of carbon corrosion in alkaline electrolytes has scarcely been reported, except for a few early reports dating back to the 1980s.^19^, ^22^

In most OER measurements catalyzed by carbon or carbon‐supported catalysts, the current measured during potentiostatic polarization is often exclusively ascribed to O_2_ evolution with carbon oxidation being presumed or shown to be negligible based on Faradaic efficiency measurements. However, at the anodic conditions of O_2_ evolution on highly active OER catalysts, O_2_ evolution and carbon oxidation are expected to proceed concurrently^23^ as depicted in Scheme 1 a. The OER occurs from a purely thermodynamic point of view at potentials higher than 1.23 V versus RHE that are far above the thermodynamic equilibrium potential of 0.207 V versus RHE^17^ of carbon oxidation. This implies that the Faradaic current measured during potentiostatic O_2_ evolution is supposedly a sum of the OER (*i*_OER_) and carbon oxidation (*i*_C,Ox_). To understand the corrosion of carbon and its implication on catalyst stability and long‐term system performance, it is important to decouple the current measured during the OER into the contributions *i*_OER_ and *i*_C,Ox_.

**Scheme 1:**
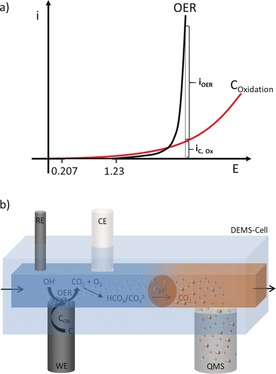
Schematic of carbon oxidation during OER in alkaline electrolytes. The current is the sum of *i*_OER_ and *i*_C,Ox_ and it is supposed that a highly active OER catalyst is protecting the carbon support against corrosion (a). Concept of CO_2_ detection as a marker for electrochemical carbon corrosion in alkaline electrolytes, for example during the OER. The formed CO_2_ is converted into CO_3_^2−^ which is again liberated as CO_2_ by injecting an acid and in turn collected through a Teflon membrane at the inlet of the MS (b).

Differential electrochemical mass spectrometry (DEMS) is a powerful technique that can be used to probe carbon corrosion and its mechanisms by direct detection of gaseous and volatile corrosion products dissolved in the electrolyte.^24^ DEMS has been used to study carbon corrosion in acidic electrolytes by direct detection of CO_2_ as a corrosion marker.^8^, ^9^, ^10^ On the other hand, direct detection of CO_2_ as a marker for carbon corrosion in alkaline electrolytes is challenging because of CO_2_ dissolution in high pH electrolytes under formation of carbonate according to Equation (4).^25^

(4) $$2\;\mathrm{OH}{}^{-}+\mathrm{CO}{}_{2}\to \mathrm{CO}{}_{3}{}^{2-}+H{}_{2}O$$

Consequently, the corrosion of carbon‐based catalysts and catalyst supports at alkaline conditions has hardly been addressed despite the broad scope of applications under these conditions. Recently, Yi et al. investigated the electrochemical stability of glassy carbon under anodic conditions in acidic and alkaline electrolyte by means of spectroscopic methods.^13^ They proposed a radical decomposition mechanism for glassy carbon at high anodic polarization in alkaline media. Edges of small graphitic domains are oxidized until they become hydrophilic and dissolve in the electrolyte.^13^

To achieve online detection of electrochemical carbon corrosion in alkaline electrolytes, we designed a DEMS cell^26^ (Figure S1 in the Supporting Information) which includes an additional channel allowing for acidification of the electrolyte without changing the electrochemical conditions at the working electrode. According to the Bjerrum plot for CO_2_, acidifying the electrolyte below a pH of 4 shifts the CO_2_/CO_3_^2−^ equilibrium completely towards CO_2_.^27^ Thus, acidifying the electrolyte will cause release of CO_2_ initially dissolved as carbonate and its subsequent detection by mass spectrometry. A schematic representation of the proposed processes is depicted in Scheme 1 b. For further information about the DEMS measurements see Supporting Information.

The proof of concept was carried out by measuring the total current density (*j*_F_, Figure 1 first row) response during potential step polarization in 0.1 m KOH (pH 12.9) of a graphite electrode, juxtaposed with the corresponding subsequently measured mass spectrometric ion currents of O_2_ (*i*_32_) and CO_2_ (*i*_44_), without (Figure 1 second and third row) and with (Figure 1 fourth row) acidification (0.15 m H_2_SO_4_, pH 0.7) of the electrolyte in front of the membrane of the DEMS system indicates that the total measured *j*_F_ is essentially the same for the two independent measurements. The independence of the electrochemical response is due to the cell design, in which acid injection and electrochemistry are spatially separated avoiding changes of the environment in front of the electrode. Evidently, the *j*_F_ represents a sum of O_2_ formation and carbon oxidation (Figure 1 first row). Without introduction of the acid, only the O_2_ ion current was detectable in the mass spectrometer. The fact that CO_2_ could not be directly detected during anodic polarization of the graphite electrode in 0.1 m KOH underlines the presence of the reaction in Equation (4). Note that the O_2_ MS signal was always recorded without acidification in order to avoid signal changes caused by the injected acid. The lack of studies on carbon corrosion in alkaline media leads to the assumption that carbon corrosion in alkaline environments occurs similarly and at comparable rates as in acidic electrolytes. According to Nernst equation, the equilibrium potential of a reaction shifts with the pH when either protons or hydroxide ions are involved in the reaction. Thus, for both the electrochemical carbon corrosion and the OER, the equilibrium potential is pH dependent. However, the kinetics of the reactions and their dependence on electrolyte pH might differ substantially. Chronopotentiometric (CP) measurements of a graphite electrode at an applied current density of 5.5 mA cm^−2^ employing electrolytes with a pH of 1 and 13 reveal a clear dependence of both the obtained potential and the measured CO_2_ ion currents on the electrolyte pH value (Figure 2 a and Figure S4). Note that acid was injected for CO_2_ release during all measurements. As expected, both acidic and alkaline environments afforded similar potentials versus RHE at the applied current density (Figure 2 a), however, the measured ion charge for CO_2_ (*Q*_44_) observed by integrating the whole ion current for CO_2_ which was produced during the measurement (Figure S4) varied substantially (Figure 2 b). The *Q*_44_ decreased from pH 1 to 13.

**Figure 1:**
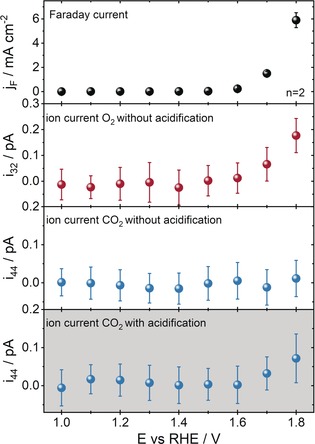
Current density response during potentiostatic polarization of a graphite rod electrode at increasing potentials (first row) in 0.1 m KOH, and the corresponding ion current for O_2_ and CO_2_, without acidification of the electrolyte (second and third row), and after acidification of the electrolyte upon injection of 0.15 m H_2_SO_4_ (fourth row). The *i*_ion_ signal for O_2_ was recorded without acidification.

**Figure 2:**
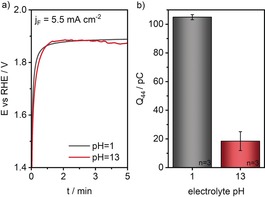
a) Chronopotentiometric measurements on graphite electrodes at an applied current density of 5.5 mA cm^−2^ in electrolytes with pH 1 and 13 (flow rate 270 μL min^−1^). b) The Electrolytes were acidified by 0.15 m H_2_SO_4_ (flow rate 270 μL min^−1^) in front of the DEMS membrane inlet in order to release the primarily formed CO_2_ present as carbonate (pH 13), and corresponding detected CO_2_ ion charge.

Comparing electrochemical carbon corrosion in electrolytes of pH 1 and 13, CO_2_ was barely detectable at high pH values, with a low CO_2_ ion charge of 18.5±6.6 pC, while it increased substantially to 104.9±1.8 pC in acidic electrolyte. This situation can be explained by the fact that during CP measurements involving competing reactions (here carbon oxidation and OER), the measured potential is closest to that of the predominant reaction. Having this in mind, the results point towards a change in the relative contributions of the OER and carbon oxidation with increasing pH value. Reconsidering Scheme 1 a, in an acidic medium the red curve would be shifted to more cathodic potential with respect to the curve of the OER and vice versa when the reaction environment is alkaline. Since the thermodynamics for both reactions in relation to the equilibrium potentials are similarly influenced by the pH value and should be invariant when referenced versus the RHE, the difference in CO_2_ detection and hence carbon oxidation, is related to a pH‐dependent change in the reaction kinetics. Obviously, the OER is kinetically favored in alkaline pH while carbon oxidation proceeds at higher rates in acidic environments. The results indicate a clear difference between carbon oxidation in alkaline and acidic conditions, indicating that a direct extrapolation of carbon corrosion from acidic conditions to alkaline environment might be misleading.

Clearly, deposition of an OER electrocatalyst on the carbonaceous electrode surface leads to an enhancement of the OER kinetics by decreasing the overpotential for the OER. Thus, a further shift in the relative contributions of the OER and carbon oxidation in favor of the OER is expected in alkaline electrolytes. It can therefore be supposed that when a sufficient coverage of an OER active catalyst is homogeneously dispersed on carbon, the tendency for carbon to undergo oxidation will be suppressed kinetically provided that the density of OER active sites is not limiting. Two model systems, Vulcan XC 72 carbon (denoted as Vulcan) and nickel boride (Ni_*x*_B), a well‐established active catalyst for the OER,^28^ supported on Vulcan XC 72 carbon (denoted as Ni_*x*_B/C‐10 for a mixture with 10 wt % Ni_*x*_B) were employed to support the aforementioned presumption.

Chronopotentiometric measurements at various current densities reveal a gradual increase of the recorded ion current of CO_2_, which was normalized by the mass of Vulcan on the electrode, with increasing applied current density when Vulcan is used as catalyst (Figure 3 black curves, for non‐normalized ion currents see Figure S5). Adding 10 wt % Ni_*x*_B to Vulcan leads to the disappearance of the mass signal of CO_2_ regardless of the applied current (Figure 3 blue curves), hence carbon oxidation is presumably suppressed by the enhanced kinetics of the OER.

**Figure 3:**
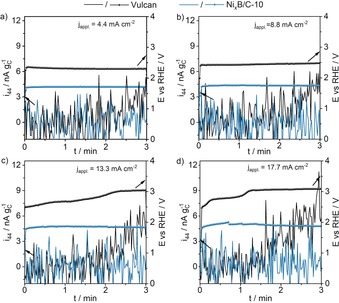
Chronopotentiometric measurements (thick lines) and ion currents (normalized by the mass of Vulcan on the electrode; lines) for CO_2_ of electrodes modified with Vulcan and Ni_*x*_B/C‐10 at applied current densities of 4.4 mA cm^−2^ (a), 8.8 mA cm^−2^ (b), 13.3 mA cm^−2^ (c), and 17.7 mA cm^−2^ (d) measured in 0.1 m KOH (flow rate 270 μL min^−1^). The electrolyte was acidified by 0.15 m H_2_SO_4_ (flow rate 270 μL min^−1^) in front of the DEMS membrane inlet.

Additionally, the potential afforded to drive the reactions at the necessary rates to fulfill the applied currents increased substantially with increasing current density in the case of pure Vulcan reaching values higher than 3 V versus RHE. The presence of several plateaus in the potential time transient of the Vulcan sample indicates that various reactions at different potentials have to proceed to provide the applied currents, while for Ni_*x*_B/C‐10 only slight changes in the potential occur. By determining *Q*_44_ from the produced CO_2_‐Signal (Figure S6) from the chronopotentiometric measurements, quantitative analysis of the CO_2_ MS signal (for calibration and carbon loss calculations see Supporting Information) reveals a Faradaic efficiency for Vulcan of 75–80 % towards CO_2_ (Figure S7) while no CO_2_ formation can be observed for Ni_*x*_B/C‐10 at any of the applied current densities. It has to be noted that despite reports suggesting that carbon oxidation in alkaline solution leads to dissolved carbonaceous molecules, in our study only CO_2_ was used as carbon oxidation marker. In addition to CO_2_, O_2_ was detected as a second reaction product (Figure S8, S9). However, Vulcan only produced a small amount of O_2_ which did not change substantially with the current density. The O_2_ MS signal detected for measurements involving Ni_*x*_B/C‐10 gradually increases with increasing current density. At the current densities that are necessary to achieve a reasonable CO_2_ MS signal, the O_2_ formation is already so vigorous that the saturation concentration of O_2_ in the electrolyte is exceeded and bubble formation hampers a proper calibration of the system for O_2_. Nevertheless, the quantitative CO_2_ data together with the qualitative O_2_ data revealed that carbon oxidation is presumably suppressed by the catalytically increased OER kinetics. Thus, supporting OER catalysts on carbonaceous materials or preparing them based on carbonaceous precursors might lead to a protection of the carbon material by the enhanced reaction kinetics provided by the catalyst at alkaline OER conditions. These findings are in agreement with the results of Lafforgue et al.^14^ on the carbon corrosion in presence of Pt. Different to Ni_*x*_B, Pt shows only minor activity for the OER, thus carbon corrosion is enhanced. Additionally, in PEMFC research it is well known that addition of the active OER catalyst IrO_2_ to the ORR catalyst hampers carbon corrosion.

Furthermore, the occurrence of a CO_2_ MS signal if catalytic activity is lost over longer time, by either catalyst deactivation or particle loss, points towards an increased carbon corrosion rate further corroborating that the presence of an OER catalyst protects carbon from oxidation even under alkaline OER conditions (Figure S10).

In conclusion, we successfully developed a new experimental DEMS‐based procedure with a unique cell design that makes it possible to directly detect CO_2_ formation as a marker for carbon corrosion in alkaline electrolytes, which has hitherto not been possible. It was demonstrated that during OER using carbon or carbon supported catalysts, OER and carbon oxidation proceed concurrently, however, carbon oxidation was considerably suppressed upon enhancing the OER kinetics using a highly active OER catalyst. Therefore, this study does not only present a new methodology for detecting carbon corrosion in alkaline electrolytes but also provides insight in the fate of carbon during electrocatalytic OER on carbon or carbon supported catalysts. The results are therefore not only valuable for fundamental understanding but are also of practical importance for monitoring carbon corrosion in technical applications.

## Conflict of interest

The authors declare no conflict of interest.

## Supporting information

As a service to our authors and readers, this journal provides supporting information supplied by the authors. Such materials are peer reviewed and may be re‐organized for online delivery, but are not copy‐edited or typeset. Technical support issues arising from supporting information (other than missing files) should be addressed to the authors.

Supplementary
