# Supplementary material for: Online Monitoring of Electrochemical Carbon Corrosion in Alkaline Electrolytes by Differential Electrochemical Mass Spectrometry
Source: Angew Chem Int Ed Engl. 2019 Dec 4;59(4):1585–9. doi: 10.1002/anie.201909475 (PMC7003769; doi:10.1002/anie.201909475)
Supplement: Supplementary file 1 — Supplementary [file ANIE-59-1585-s001.pdf]

Angewandte Chemie
Eine Zeitschrift der Gesellschaft Deutscher Chemiker

Supporting Information

Online Monitoring of Electrochemical Carbon Corrosion in Alkaline Electrolytes by Differential Electrochemical Mass Spectrometry

*Sandra Möller, Stefan Barwe, Justus Masa, Daniela Wintrich, Sabine Seisel, Helmut Baltruschat, and Wolfgang Schuhmann**

anie_201909475_sm_miscellaneous_information.pdf

Experimental part

Chemicals and materials

All chemicals were of analytical grade and used as received unless explicitly stated otherwise. Potassium hydroxide pellets were from Carl Roth (Karlsruhe, Germany), sulfuric acid (>95%) was from Fisher (Loughborough, United Kingdom), ethanol and Nafion® 117 solution were from Sigma Aldrich (Steinheim, Germany) and potassium hydrogen carbonate was from Sigma Aldrich (Steinheim, Germany). The used water (MilliQ) was purified by a water purification system (SG Water, Germany) and had a conductivity of 0.055 µS cm^{-1}. High-purity Argon was from AirLiquide (Dortmund, Germany). Vulcan XC-72 was a donation from Cabot Corporation and nickel boride was synthesized according to the method described by Masa et. al.[1] Glassy carbon rods were from HTW (Thierhaupten, Germany) and graphite rods were from Ringsdorff-Werke (Bad Godesberg-Mehlem, Germany).

Electrolytes

For all measurements, the electrolytes were purged with high-purity Ar. General: 0.1 M KOH (pH=12.9) and 0.15 M H_2SO_4 (pH=0.7) were prepared using KOH pellets/H_2SO_4 (>95%) and water. The pH of the electrolyte of pH=1 was adjusted by mixing 0.1 M KOH and 0.15 M H_2SO_4. Potassium hydrogen carbonate ($KHCO_3$) was added to 0.1 M KOH to obtain solutions with 40, 50, 75, 100 and 125 mg·L^{-1} $KHCO_3$. The electrolytes were stored in glass bottles and under argon atmosphere.

Electrode preparation

The glassy carbon electrodes (A=0.113 cm^{-2}) were drop-coated with a specific volume of a catalyst suspension (1. c_{Vulcan} = 5.0 mg mL^{-1} and 2. c_{NixB} = 5.0 mg mL^{-1} in a solvent mixture of Nafion® + ethanol + water (volume ratio 2:49:49)) to obtain an initial catalyst loading of 210 µg cm^{-2}. Prior to use, the catalyst suspensions were sonicated in a sonication bath for 15 min to obtain a stable and homogeneous suspension. The suspensions (1. and 2.) were mixed to obtain suspensions with a Ni_xB content of 10 wt%. After mixing the suspensions, they were additionally sonicated in a sonication bath for 5 min. Prior to use, the electrodes were polished with a polishing cloth (LECO) with alumina pastes of different grain sizes (3.0, 1.0, 0.2 and 0.05 micron sizes) in order to get a mirror like electrode surface.

The graphite electrodes with a surface area of 0.785 cm^{-2} (active surface area A=0.361 cm^{-2}; the active surface area is determined from the channel geometry of the DEMS-cell configuration) were used as obtained and the electrodes with a surface area of 0.283 cm^{-2} (active surface area* A=0.228 cm^{-2}) were polished with polishing paper with a grain size of 3 µm, 1 µm and 0.3 µm (3M, Germany). Prior to use, the electrodes were rinsed with water.

Electrochemistry

All the electrochemical measurements were performed at room temperature (RT) in a custom-built DEMS cell (see DEMS-cell configuration) with either the catalyst modified glassy carbon electrodes or the graphite rod electrodes as working electrode (WE), a Ag/AgCl/3 M KCl as reference electrode (RE) and a platinum mesh as counter electrode (CE). The CE was separated from the WE volume by a ceramic frit to prevent crossover of products generated at the CE to the WE compartment. Potentiostatic and galvanostatic measurements were performed using an Autolab PGSTAT 204 (Metrohm-Autolab, The Netherlands) controlled by the NOVA 2.0 software. All potentials are referenced against the reversible hydrogen electrode (RHE) according to Equation S1:

$$E_{RHE} = E_{appl} + E^0_{Ag/AgCl} + 0.059\ pH \qquad \text{Eq.S1}$$

Where $E_{Ag/AgCl}$ is the potential with respect to the Ag/AgCl/3 M KCl and $E^0_{Ag/AgCl}$ (0.207 V) is the potential of the Ag/AgCl/ 3 M KCl electrode with respect to the standard hydrogen electrode (SHE) at standard conditions. The pH of 0.1 M KOH (pH of 12.8) was measured with a pH electrode for highly alkaline solutions (Dr. Kornder, Germany). The potential was further corrected for uncompensated electrolyte resistance according to $E^{corr}_{RHE} = E_{RHE} - i \cdot R$. The uncompensated resistance was determined by means of electrochemical impedance spectroscopy (EIS) using an *ac* perturbation of 10 mV (rms) at open circuit potential in the frequency range from 20 kHz to 1 Hz.

Differential electrochemical mass spectrometry (DEMS)

The ion currents for the mass of carbon dioxide (CO_2, m/z = 44) and oxygen (O_2, m/z = 32) were detected by means of differential electrochemical mass spectrometry (DEMS) in parallel to the electrochemical measurements. The mass spectrometer is a GAM 400 from InProcess Instruments (Bremen, Germany). The used porous Teflon membrane (GoreTex) has a pore width of 20 nm and a thickness of 75 µm. A schematic representation of the custom-built DEMS cell is depicted in Figure S1. The cell body made of polymethyl methacrylate (PMMA) is coupled to the mass spectrometer via a stainless steel flange. A porous hydrophobic Teflon membrane prevents the electrolyte from entering the MS. To withstand the pressure difference between the vacuum system and the liquid phase, the Teflon membrane is supported by a polyetheretherketone (PEEK) frit. The three-electrode-setup (WE, RE and CE) is at the electrolyte inlet (Figure S1a), whereas the Teflon membrane is located at the electrolyte outlet (Figure S1b). Between the main reaction volume of the cell and the Teflon membrane, there is an additional electrolyte inlet (Figure S1c). Furthermore, there is an optional screw (Figure S1d) counterpart of the WE to release gas bubbles. The channel below the working electrode is 3.8 mm wide and 3.0 mm in height. Between the CE and the gas entrance to the mass spectrometer (MS), a reduction in the channel height is introduced to facilitate electrolyte mixing. The decrease is from 3.0 mm to 1.0 mm in height. The holes through which the electrolytes are introduced to the cell have a diameter of 2.0 mm.

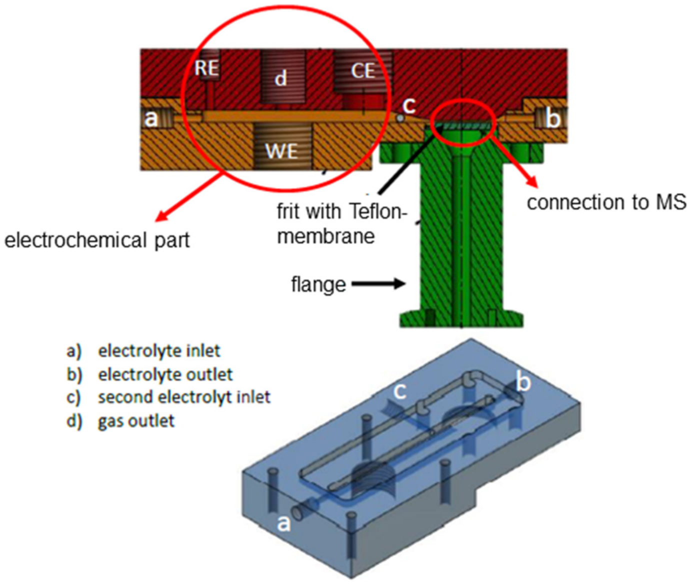

Figure S1. Schematic representation of the DEMS-cell configuration.

Potential step measurements

The described concept to detect CO_2 as a carbon corrosion marker in alkaline electrolytes was tested by potential step polarization of a graphite electrode (active surface area = 0.228 cm^{-2}) in a potential window of 1.0 V to 1.8 V vs RHE with potential steps of 100 mV for 300 s. In parallel to the potentiostatic measurement, the ion currents i_{32} and i_{44} for O_2 and CO_2 were detected by means of DEMS. The electrolyte during the electrochemical reactions was 0.1 M KOH saturated with high-purity Ar at a flow rate of 270 µL min^{-1}. 0.15 M H_2SO_4 was introduced into the cell through the additional electrolyte entrance with a flow rate of 270 µL min^{-1} in order to acidify the electrolyte for CO_2 detection. For O_2 detection 0.1 M KOH was introduced to the cell through the additional electrolyte entrance at a flow rate of 270 µL min^{-1}. The average faraday current density and ion current signals detected by MS for the last 100 s were evaluated and plotted against the applied potential.

Detection of CO_2 from acidic and alkaline electrolytes by means of DEMS

The detection of CO_2 from the electrolyte by the presented DEMS system is based on the presumption that the CO_2/CO_3^{2-} equilibrium is fully shifted to CO_2 at pH values below 4. In order to achieve the necessary pH shift. When alkaline electrolyte is used for the measurements, H_2SO_4 (0.15 M, pH 0.7) is injected via the aforementioned side inlet of the DEMS cell. The injection of the acid leads to a change in the pH value of the electrolyte to a value of 1.3 before it passes the DEMS membrane inlet. Hence, all potentially formed carbonate is supposedly released as CO_2 and detectable by DEMS. For quantitative analysis, the DEMS system was calibrated with carbonate solutions in a range of theoretically expectable

concentrations taking into account different faradaic efficiencies for carbon oxidation at various applied currents (for a detailed description of the DEMS calibration see the related section below). In order to avoid errors due to inhomogeneous mixing of the two solutions and the formed carbonate in the electrolyte, the calibration was done at steady state conditions ensuring that the detected CO_2 resembles the used concentration, while the detection of the electrochemically formed CO_2 was done in a time span beginning with the start of the electrochemical procedure until the CO_2 DEMS signal went as low again as the baseline ensuring detection of all formed and released CO_2. Moreover, even assuming a non-equal product distribution in front of the working electrode, the initial CO_3^{2-} and after acid injection the CO_2 concentration may be higher than upon equal mixing, and hence, the detected amount of CO_2 is the upper limit.

Carbon oxidation in dependence of electrolyte pH

Galvanostatic measurements on a graphite electrode (active surface area = 0.190 cm^{-2}) at a current density of 5.5 mA cm^{-2} for 5 min were done in order to analyze the carbon oxidation in dependence of the electrolyte pH value. Electrochemical measurements were conducted in Ar-saturated electrolytes with pH values of 1and 13 at a flow rate of 270 µL min^{-1}. In parallel to the galvanostatic measurements, the ion current for CO_2 (i_{44}) was measured by DEMS (Figure S4). For CO_2 detection, 0.15 M H_2SO_4 (flow rate 270 µL min^{-1}) was injected through the additional electrolyte entrance of the cell (Figure S1c). The ion charge for CO_2 (Q_{44}) measured during the chronopotentiometric measurements (Figure 2a), was obtained by integrating the whole ion current peak areas (Figure S4).

Carbon oxidation on Vulcan and Ni_xB/C-10

Carbon oxidation was studied on a glassy carbon electrode (active surface area 0.113 cm^{-2}) drop-coated with two model catalysts, Vulcan XC 72 carbon (denoted as Vulcan), and a loading of nickel boride (Ni_xB) supported on Vulcan XC 72 carbon (denoted as Ni_xB/C-10 for a mixture with 10 wt.% Ni_xB, respectively). The electrode preparation is described under electrode preparation.

The electrochemical carbon oxidation behavior of Vulcan and Ni_xB/C-10 was studied by chronopotentiometry (CP) measurements at current densities of 4.4, 8.8, 13.3 and 17.6 mA cm^{-2} (Figure 3 a-d and S7 a-d, sphere) for 3 min in 0.1 M KOH saturated with high-purity Ar, with a flow rate of 270 µL min^{-1}. In parallel to the CP measurements, the ion currents for CO_2 (i_{44}) and O_2 (i_{32}) were measured by mass spectrometry (Figure 3 a-d and S7 a-d, straight line). To detect CO_2 the KOH has to be acidified. Therefore, 0.15 M H_2SO_4 (Ar saturated) was injected through the additional electrolyte entrance of the cell (Figure S1c) with a flow rate of 270 µL min^{-1}. To detect O_2 the KOH was not acidified. To have the same conditions, 0.1 M KOH (Ar saturated) was injected through the additional electrolyte entrance of the cell with a flow rate of 270 µL min^{-1}. The detected ion currents for CO_2 (i_{44}) were normalized with

respect to the carbon content in the catalyst sample based on the amount of Vulcan. Figure S5 show the whole ion current peak for CO_2 (i_{44}), which arise from the before described CP measurements. For non-normalized ion currents see figure S5. The ion charge for CO_2 (Q_{44}) measured during the chronopotentiometric measurements (Figure 3, S5), was obtained by integrating the whole ion current peak areas (Figure S6). With the Q_{44} and the obtained equation from the subsequent descript calibration curve (Figure S3) the FE_{CO2} (Eq. S2, Figure S7) was calculated. To obtain the ion charge for O_2 (Q_{32}, Figure S9) the ion current for O_2 (i_{32}, Figure S8) was integrated for the acquisition time.

DEMS calibration for CO_2

The DEMS was calibrated for CO_2 by recording the ion current of CO_2 (i_{44}) using 0.1 M KOH with various known (see electrolyte fabrication) concentrations of $KHCO_3$. 0.15 M H_2SO_4 was constantly flowing through the additional electrolyte inlet in order to release CO_2 from the dissolved carbonate. Both electrolytes had a flow rate of 270 $\mu L \cdot min^{-1}$. For later analysis only ion currents which emerge from the detection limit were evaluated. The detection limit is defined as $3 \cdot \delta$, where δ is the standard deviation of the average baseline for the CO_2 ion signal (i_{44}). In this case i_{44} is 0.11 pA. To determine the ion charge for CO_2 (Q_{44}), the last 10 minutes before changing the solution were integrated as shown in Figure S2.

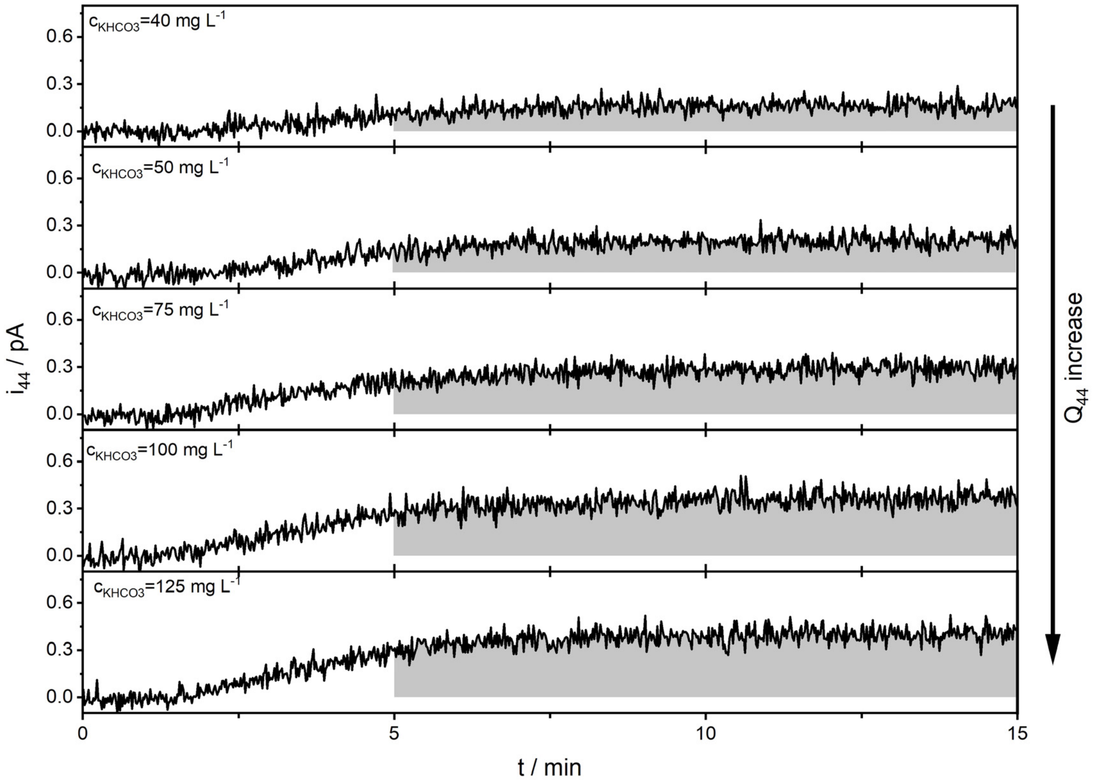

Figure S2. Mass spectrograms for the CO_2 ion currents (i_{44}) of solutions with various $KHCO_3$ concentrations in 0.1 M KOH (flow rate 270 $\mu L\ min^{-1}$). To enable CO_2 detection, 0.15 M H_2SO_4 was injected into the cell through the additional electrolyte entrance (flow rate 270 $\mu L\ min^{-1}$). Integrated area (grey) to obtain the ion charge for CO_2 (Q_{44}).

Plotting Q_{44} against the carbonate concentration (c_{KHCO3}) leads to a linear calibration curve (Figure S3).

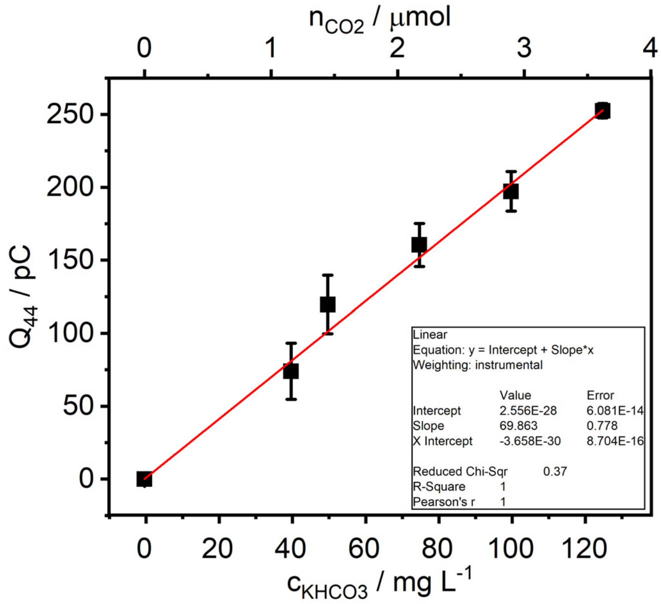

Figure S3. Calibration graph with the linear fit (red line) from which the calibration was obtained.

With the linear equation of the calibration curve, the measured ion charges for CO_2 (Q_{44}) during electrochemical experiments were correlated to a produced CO_2 content (n_{CO2}). The Faradaic efficiency for CO_2 (FE_{CO2}) was calculated according to Equation S2, with n_F being the amount of theoretically producible CO_2 at the applied specific faradaic charges (assuming a theoretically faradaic charge to CO_2 conversion of 100 %).

$$FE_{CO2}\ [\%] = \frac{n_F \cdot 100\%}{n_{CO2}} \quad \text{Eq.S2}$$

Accelerated stress test on Ni_xB/C-10

An accelerated stress test was applied to a Ni_xB/C-10 modified electrode. The stress test consists of a sequence of electrochemical measurements as depicted in Scheme S1. The measurements were done in 0.1 M KOH while 0.15 M H_2SO_4 was introduced to the additional electrolyte entrance to guarantee CO_2 release. Both electrolytes had a flow rate of 270 µL min^{-1}. To activate the catalyst five conditioning CVs in a potential range of 1.0 to 1.7 V vs RHE with a scan rate of 50 mV s^{-1} were applied. Afterwards CV-measurements in a potential range of 1.0 to 2.0 V vs RHE at a scan rate of 50 mV s^{-1} were done before and after each CP-measurements. Current densities of 26.5, 35.4 and 44.2 mA cm^{-2} were applied during the chronopotentiometric measurements. During the whole test the ion current for CO_2 (i_{44}) was detected.

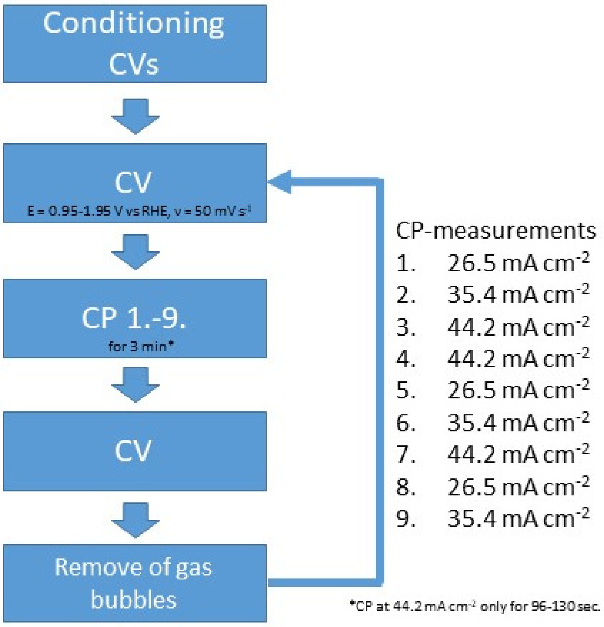

Scheme S1. Schematic representation of the measurement sequence of the accelerated electrode stress test on Ni_xB/C-10.

Additional results and discussion

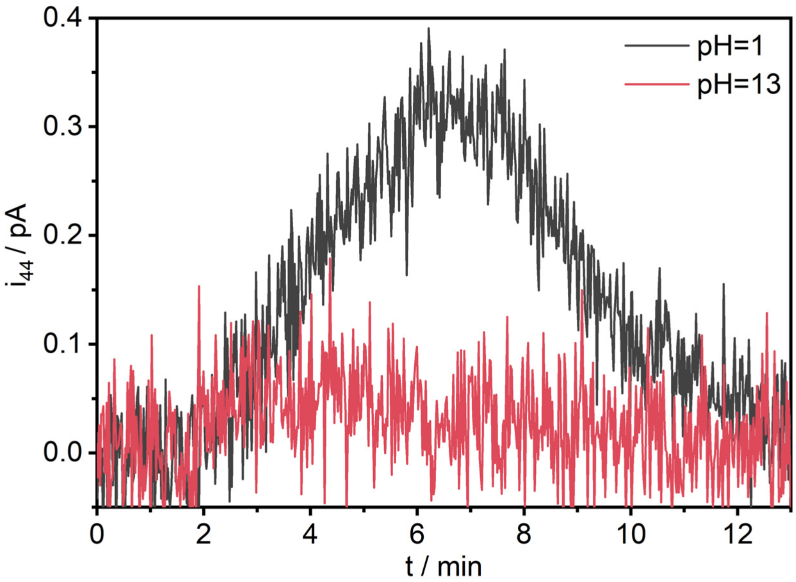

Figure S4. Ion currents for CO_2 (i_{44}) in electrolytes of pH 1 and pH 13 (flow rate 270 µL min^{-1}) recorded during chronopotentiometric measurements at 5.5 mA cm^{-2} (Figure 2a). To enable CO_2 detection, 0.15 M H_2SO_4 was injected into the cell through the additional electrolyte entrance (flow rate 270 µL min^{-1}).

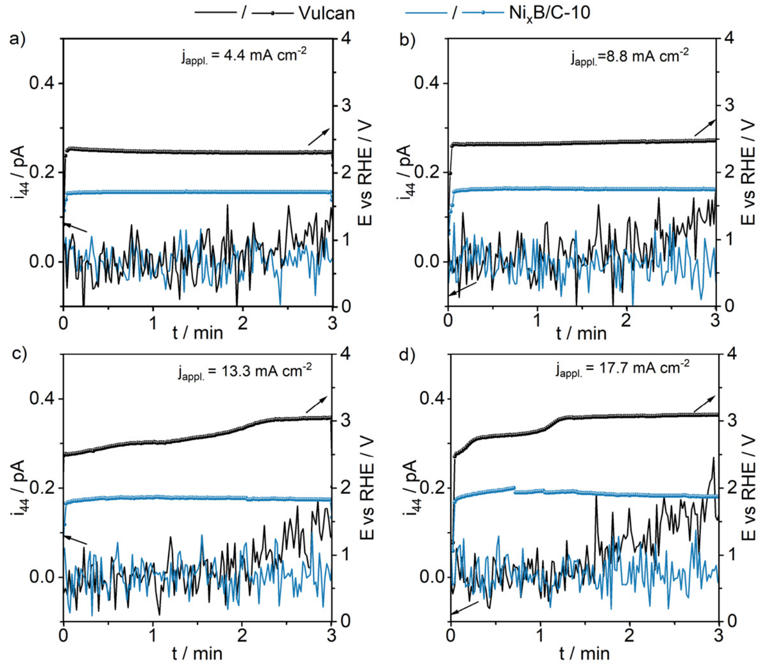

Figure S5. Chronopotentiometric measurements (spheres) and ion currents signals (lines) for CO_2 of electrodes modified with Vulcan and Ni_xB/C-10 at an applied current density of 4.4 mA cm^{-2} (**a**), 8.8 mA cm^{-2} (**b**), 13.3 mA cm^{-2} (**c**) and 17.7 mA cm^{-2} (**d**). 0.1 M KOH flow rate 270 µL min^{-1}; H_2SO_4 (0.15 M) was injected into the electrolyte at a flow rate of 270 µL min^{-1} through a side entrance of the cell.

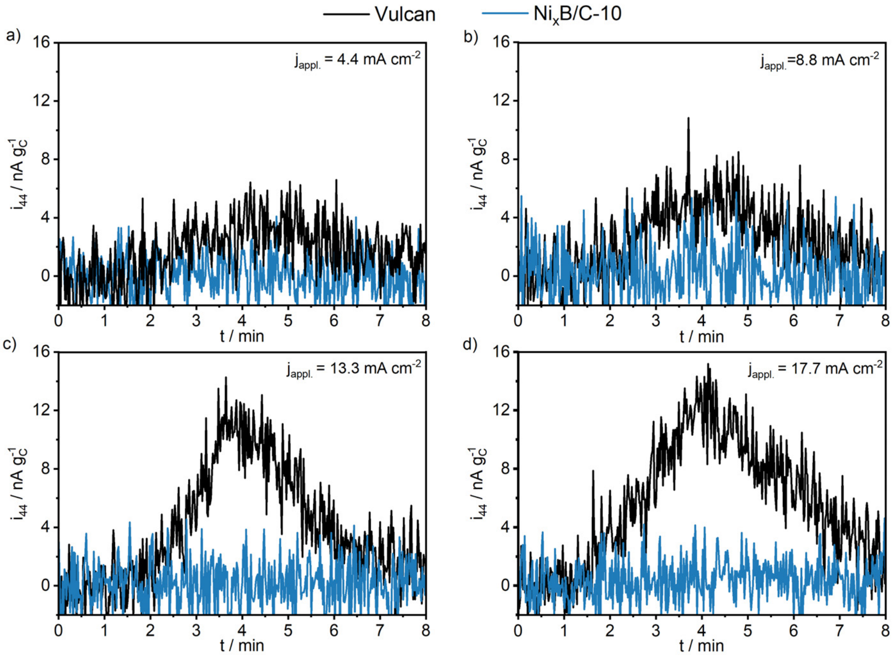

Figure S6. Mass spectrograms of the whole ion currents for CO_2 (i_{44}) recorded during chronopotentiometric measurements (Figure 3, S5) of electrodes modified with Vulcan and Ni_xB/C-10 in 0.1 M KOH (flow rate 270 µL min^{-1}). To enable CO_2 detection, 0.15 M H_2SO_4 was injected into the cell through the additional electrolyte entrance (flow rate 270 µL min^{-1}).

The amount of carbon loss during chronopotentiometric measurements at different applied current densities is summarized in Table S1. The electrodes with a loading of 210 µg cm^{-2} contain 23.73 µg Vulcan. Using the ion charge, it was possible calculate the produced mol of CO_2 (n_{CO2}) from Figure S3. The ratio between the formed CO_2 and loss of carbon (C) from the electrode surface is 1:1. It has to be mentioned that the formed CO_2 during the CP-measurement at a current density of 4.4 mA cm^{-2} is below the detection limit of the ion current of CO_2 (see section DEMS calibration for CO_2).

Table S1: Calculation of carbon loss during chronopotentiometric measurements at varying current densities.

J / mA cm^{-2}	Q_{44} / pC	n_{CO2} / µmol	$m_{C,loss}$ / µg
8.8	26.52±6.03	0.38±0.09	4.56±1.04
13.3	36.39±7.42	0.52±0.11	6.24±1.32
17.7	53.04±7.97	0.76±0.11	9.12±1.32

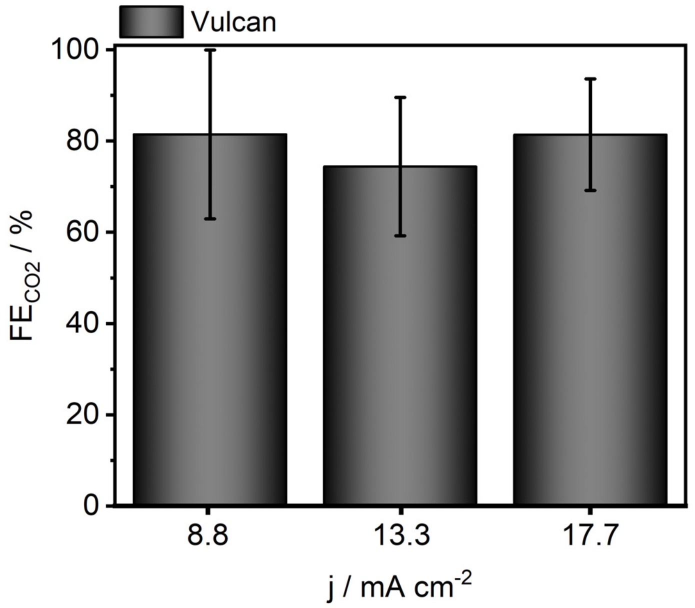

Figure S7. Faradaic efficiencies of a Vulcan-modified electrode towards CO_2 formation at different applied current densities.

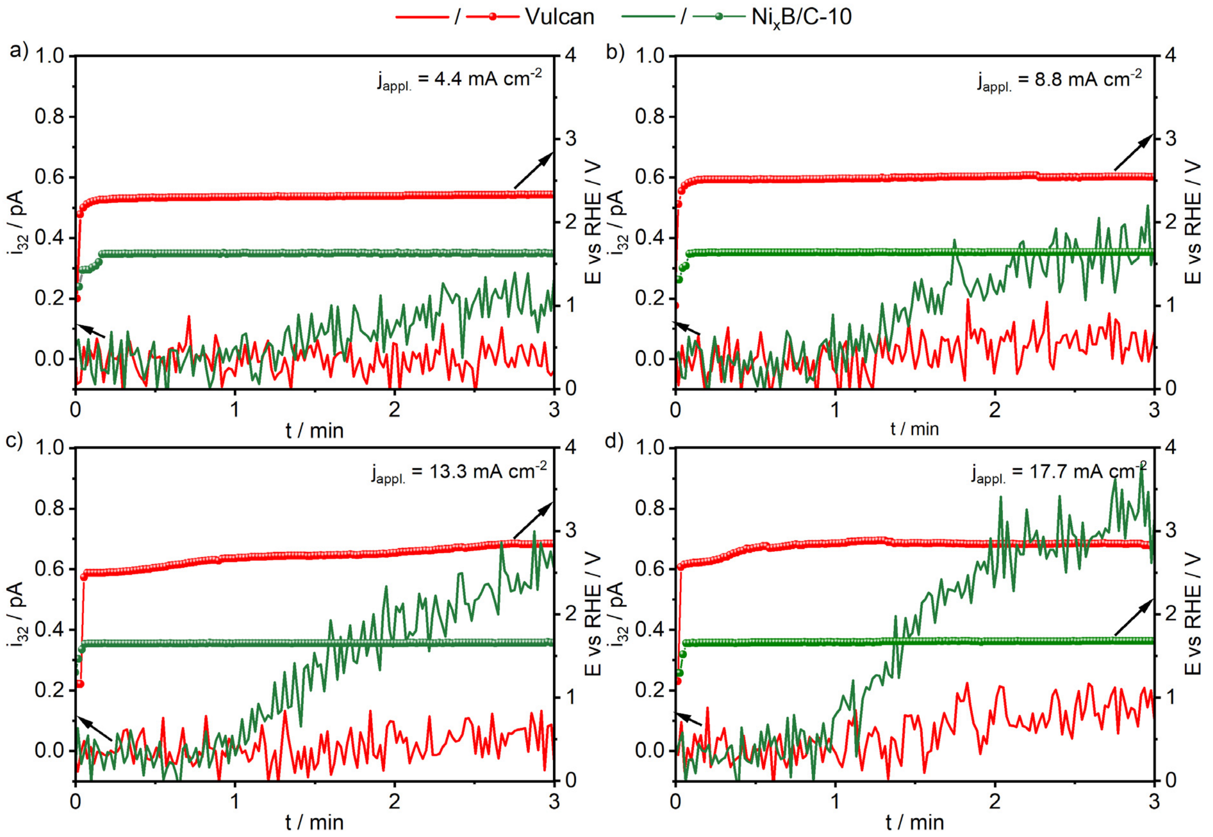

Figure S8. Chronopotentiometric measurements (spheres) and ion currents (lines) for O_2 of electrodes modified with Vulcan and Ni_xB/C-10 at an applied current density of 4.4 mA cm^{-2} (a), 8.8 mA cm^{-2} (b), 13.3 mA cm^{-2} (c) and 17.7 mA cm^{-2} (d) in 0.1 M KOH (flow rate 270 µL min^{-1}). 0.1 M KOH was injected into the cell through the additional electrolyte entrance (flow rate 270 µL min^{-1}).

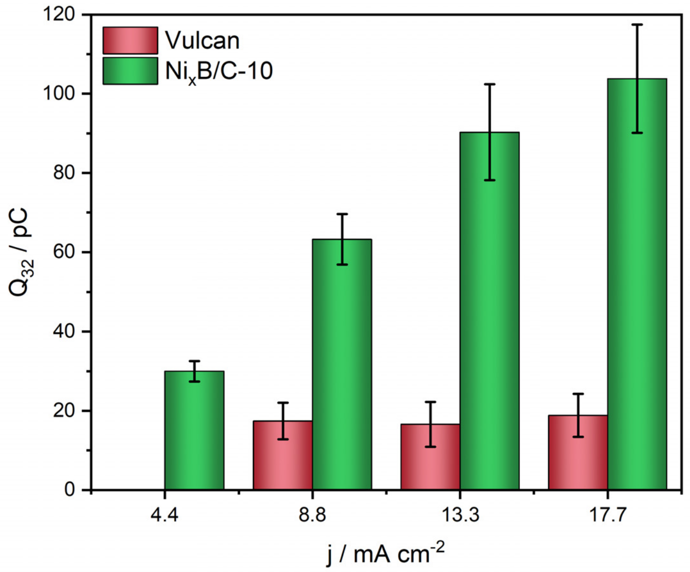

Figure S9. Ion charge for O_2 (Q_{32}) vs. applied current density for electrodes modified with Vulcan and Ni_xB/C-10.

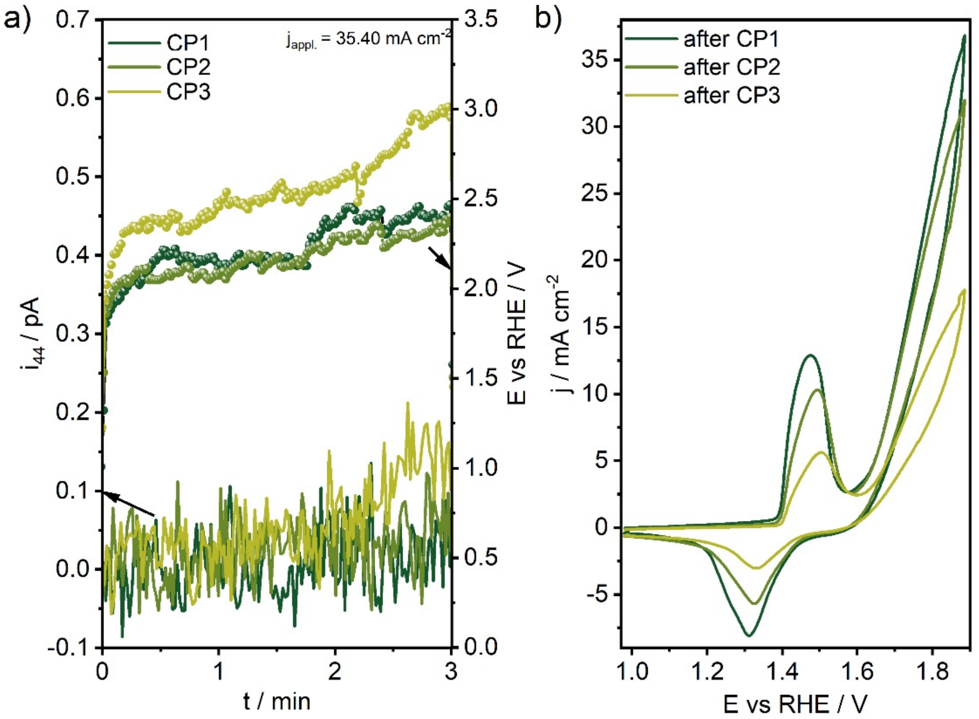

Figure S10. Mass spectrograms of a Ni_xB/C-10 modified electrode recorded during the chronopotentiometric measurements of the stress test sequence with an applied current density of 35.4 mA cm^{-2} in 0.1 M KOH (flow rate 270 µL min^{-1}) (a). To enable CO_2 detection, 0.15 M H_2SO_4 was injected into the cell through the additional electrolyte entrance (flow rate 270 µL min^{-1}). Cyclic voltammograms of aforementioned electrodes after the chronopotentiometric measurement at an applied current density of 35.4 mA cm^{-2} in 0.1 M KOH (flow rate 270 µL min^{-1}) (b).

Applying the aforementioned accelerated stress test to a Ni_xB/C-10 modified electrode it becomes obvious that the OER activity is decaying over the duration of the measurements. This decay in OER activity is most likely due to loss of active Ni_xB particles, which is represented in a decrease in the $Ni^{2+/3+}$ redox signal shown in the CVs (Figure S10b). When the amount of active catalyst becomes too small to fulfill the applied current density by the OER, carbon oxidation steps in as it can be seen in the mass spectrograms during the chronopotentiometric measurements (Figure S10a). The loss of fewer particles as after the 2nd CP is not critical since no CO_2 was detected by MS. However, is the catalyst decreasing further, CO_2 becomes to be detectable by MS, hence carbon oxidation takes place.

Reference

[1] J. Masa, I. Sinev, H. Mistry, E. Ventosa, M. d. La Mata, J. Arbiol, M. Muhler, B. Roldan Cuenya, W. Schuhmann, *Adv. Energy Mater.* **2017**, *7*, 1700381.
